# Resection of Retroperitoneal Extra-Adrenal Pheochromocytoma: A Case Report

**DOI:** 10.7759/cureus.43185

**Published:** 2023-08-09

**Authors:** Imad Ghantous, Aziz Najjar, Yehya Tlaiss, Samer Danaf, Camil J Chouairy

**Affiliations:** 1 Urology, Saint George University Hospital in Beirut, Beirut, LBN; 2 Urology, University of Balamand, Beirut, LBN; 3 Pathology, Saint George University Hospital in Beirut, Beirut, LBN

**Keywords:** multidisciplinary management, double j-stent, surgical resection, retroperitoneal tumor, extra-adrenal pheochromocytomas

## Abstract

Extra-adrenal pheochromocytomas are rare catecholamine-producing tumors that arise from chromaffin cells outside the adrenal glands. We report on the case of a 62-year-old male who initially presented with upper respiratory tract symptoms and was found to have a suprapubic pelvic mass and an asymptomatic right inguinal hernia. The diagnostic evaluation involved an abdominal ultrasound, a CT scan, followed by an MRI, which revealed a well-marginated large mass whose characteristics indicated a retroperitoneal sarcoma. Upon successful surgical resection, the mass was found to be encapsulated and no peripheral structure invasion was present; the right inguinal hernia was repaired, and a double J-stent was placed. Histopathological examination revealed extra-adrenal pheochromocytoma. This case report sheds light on diagnostic and therapeutic challenges when dealing with extra-adrenal pheochromocytomas and the importance of considering them as a differential diagnosis when presented with a case of retroperitoneal mass.

## Introduction

Pheochromocytomas are uncommon neoplasms of the adrenal medulla that originate from cells of the neuroectoderm. When these neoplasms are located outside the adrenal medulla, they are called extra-adrenal pheochromocytomas [1]. The World Health Organization’s meticulous classification, which is primarily guided by pathologists, exclusively classifies pheochromocytomas only as adrenal medullary tumors and identifies all other locations with the term paraganglioma [2]. In the context of this case, the definition put forth by Neumann is employed for practicality [3]. Here, the term pheochromocytoma is used for adrenal and extra-adrenal (15%) abdominal, thoracic, and pelvic tumors, most of which exhibit hormonal activity. On the other hand, the term paraganglioma is exclusively utilized primarily for tumors located in the head and neck area [3]. These tumors are rare neuroendocrine tumors that can emerge from chromaffin cells situated in various locations within the body. The specific site of origin significantly impacts how these tumors manifest clinically and how they are treated. For instance, extra-adrenal pheochromocytomas have been documented in areas like the head, neck, chest, and even within the walls of the bladder [4]. These tumors are mostly hormone-active, meaning they can lead to the overproduction of catecholamines, which causes symptoms such as hypertension, headache, palpitations, diaphoresis, tremors, and panic attacks [5]. However, the prevalence of pheochromocytoma is 0.1-0.6% in the hypertension outpatient clinic [6]. Additionally, extra-adrenal pheochromocytomas may arise sporadically or may be inherited in about 25% of cases [2]. Hereditary forms of extra-adrenal pheochromocytomas include von Hippel-Lindau disease (VHL), neurofibromatosis type 1 (NF1), and multiple endocrine neoplasia type 2 (MEN 2). There are currently 10 predisposing genes associated with the aforementioned forms of inheritance. This is particularly important to take into consideration when assessing a retroperitoneal mass if the patient has any of the above hereditary diseases. Most diagnostic and management difficulties are due to extra-adrenal pheochromocytomas’ rare occurrence, approximately 0.8 cases per 100,000 person-years [5], and anatomical location outside the adrenal glands. The lack of early, specific symptoms often results in a delayed diagnosis and larger tumor sizes. Most symptoms and clinical signs are a result of the direct actions of secreted catecholamines, e.g., hypertension, diaphoresis, tachycardia, and panic attacks. However, there have been cases of extra-adrenal pheochromocytomas emitting an equal amount of epinephrine and norepinephrine, which leads to normal blood pressure in such patients [6]. Hence, we cannot rely solely on clinical manifestations to identify these neoplasms. Imaging techniques, such as CT and MRI, play a crucial role in visualizing these tumors; however, their histological classification remains challenging, with accurate identification typically achieved through surgical resection. In this case report, we aim to provide insight into the diagnostic process and surgical management of an extra-adrenal pheochromocytoma while emphasizing the importance of complete resection and post-op surveillance.

## Case presentation

We describe a 62-year-old male patient with a history of atrial fibrillation, dyslipidemia, coronary artery disease, and tachycardia who presented initially to a peripheral hospital for bronchitis, at which time an abdominal ultrasound was done for chronic abdominal pain. He was revealed to have a suprapubic, heterogeneous, hyperechoic mass measuring 9.3 cm × 8 cm. This was followed by a CT scan, which identified a suprapubic pelvic mass measuring 9.5 cm × 9.2 cm × 8.5 cm with central necrosis and curvilinear calcifications with no gross adjacent vascular or organ invasion. A right inguinal hernia was detected, which is a potential finding accompanying retroperitoneal masses of this size. The patient was then presented to our care, where an MRI was ordered for better identification and assessment of organ invasion. A large, well-marginated mass was described, with evidence of hypercellularity without a definite origin in solid organs. Furthermore, soft tissue and fat were found, with findings highly suggestive of retroperitoneal sarcoma. The patient otherwise denied any prodromal symptoms, with a review of systems and a physical exam only pertinent for a palpable mass in the infraumbilical abdomen. No biopsy was done, as it is contraindicated due to the high suspicion of retroperitoneal sarcoma [7].

**Figure 1:**
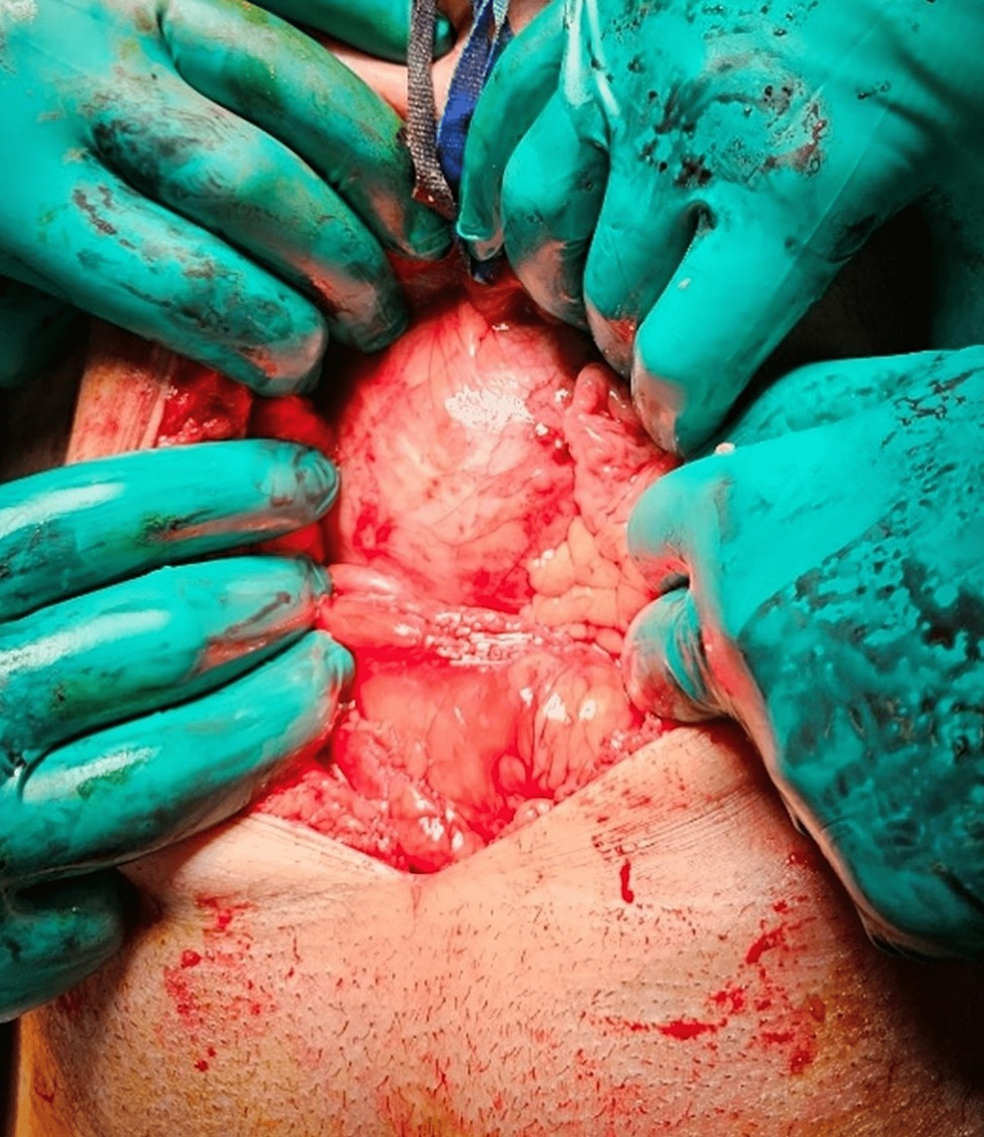
Tumor during dissection

**Figure 2:**
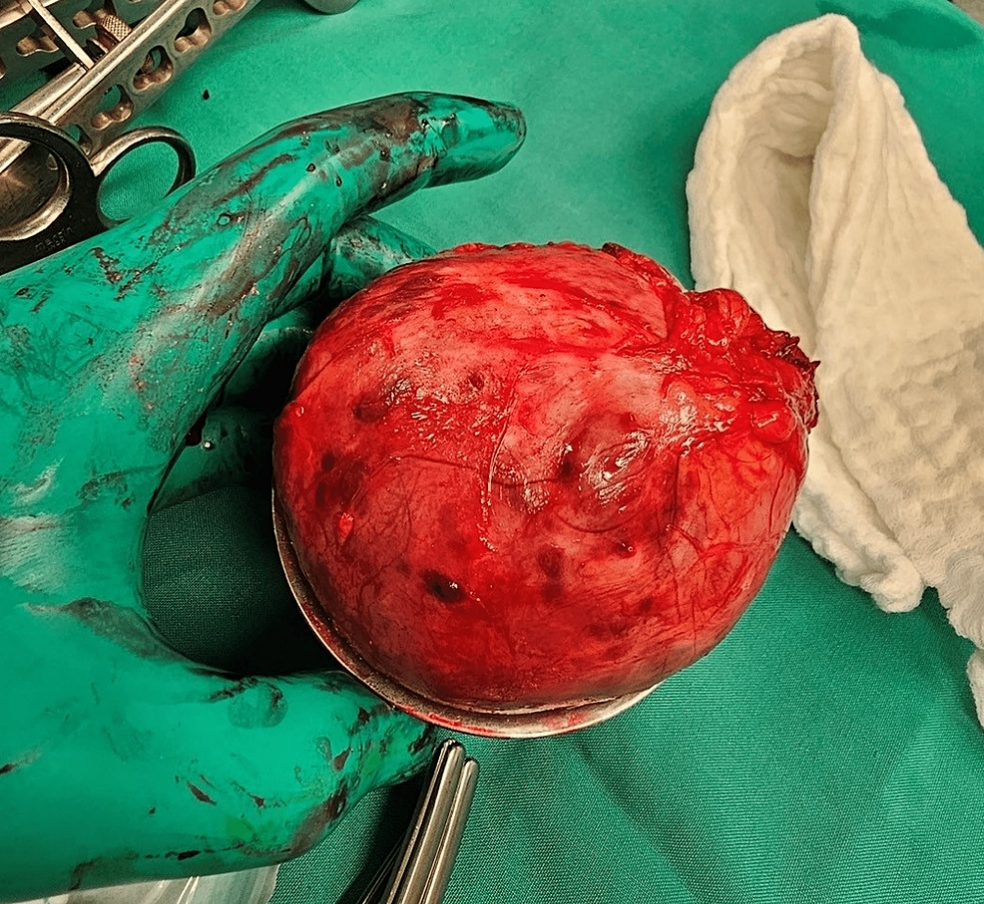
Specimen after resection

Surgical resection was chosen, and it was done through an infraumbilical incision, followed by dissection in layers that ended in access to the peritoneum, at which point the intestines were mobilized and the mass was revealed (Figure 1). Using fine dissection and Ligature, tumor attachments were gradually resected, with no peripheral structure invasion noted (Figure 2). Following the removal of the tumor, a right inguinal hernia was also repaired with mesh placement. A bilateral double J-stent was also placed to prevent ureteral obstruction and provide support during the healing process [8]. No significant intraoperative blood pressure variations were noted. Postoperatively, the patient had normal blood pressure, and he was discharged home on postoperative day 3 after monitoring. The pathology report confirmed the diagnosis of a 10 cm × 8 cm × 8 cm extra-adrenal pheochromocytoma with a GAPP score of 5/10. Resection margins were clear, including four benign lymph nodes that were negative for metastasis. On day 8 post-op, the double J-stent was removed, and the patient was followed as an outpatient.

## Discussion

This case report highlights the diagnostic and therapeutic challenges associated with extra-adrenal pheochromocytomas. The successful surgical management of this neoplasm, achieved through complete resection and meticulous dissection, underscores the importance of a multidisciplinary approach involving radiology, pathology, and surgical expertise. Accurate preoperative imaging and careful intraoperative dissection are necessary for achieving optimal outcomes and minimizing the risk of intraoperative complications. Finally, postoperative surveillance should not be overlooked when it comes to extra-adrenal pheochromocytomas.

Given the rarity of this tumor in the pelvic area, it is important to report on this case to enrich the existing literature. However, in another case report by Imperatore et al. [9], a 69-year-old guy with a mass emerging from the pelvic sidewall was determined to have an extra-adrenal pheochromocytoma that was nonfunctional. This further supports the fact that the majority of occurrences of these tumors originating within the pelvic region are not associated with hormone production, and the identification of these cases typically occurs during later stages, prompted by vague symptoms or unintended discovery [9].

Intraoperative preparation and management choices are essential when dealing with a potential catecholamine-secreting tumor. A meta-analysis performed by Hariskov and Schumann [10] on the intraoperative management of patients with incidental catecholamine-producing tumors highlighted hemodynamic instability, which was described as hypertensive episodes, as the main complication present in all cases except one [10]. In 45 of the 62 cases, specific medication for the treatment of hemodynamic instability was described. These medications were between one and six different vasoactive agents to treat hypertension. In our case, with a patient who showed no family history or typical symptoms of extra-adrenal pheochromocytomas, our operating room was equipped in case of a hypertensive event. However, even in medically prepared patients, there is a 25% chance of sustained hypertensive events occurring during surgery [10]. Hence, our case report should inform clinicians about the potential risks associated with retroperitoneal masses and the possibility of them being extra-adrenal pheochromocytomas.

Surveillance of the patient will be crucial to monitor for any signs of hypertension exacerbated by the catecholamine crisis [10]. Moreover, the placement of a bilateral double J-stent is an important step after the removal of a retroperitoneal mass of this size for ureteral obstruction prevention, protection, and support of the ureters. Additionally, long-term follow-up is important to screen for any signs of tumor recurrence. Due to the high prevalence of familiar syndromes (around 25%) in patients presented with pheochromocytomas, it is beneficial to screen for genetic mutations even in those with no known family history [2]. Furthermore, we encourage family members to get screened if a germline mutation is detected.

It is recommended that all patients be followed up every year for a minimum of ten years post-surgery, and patients with extra-adrenal pheochromocytoma be followed up lifelong [2]. In the case of genetic testing being negative, recurrence is highly unlikely. Regular imaging studies, such as CT, and clinical and biological examinations, such as measurement of urinary catecholamines, should be conducted at appropriate intervals to evaluate and detect any residual or recurrent tumor.

## Conclusions

In conclusion, this case report provides valuable insights into the surgical management and follow-up of an extra-adrenal pheochromocytoma, especially in the case of retroperitoneal masses. The clinical case reported here is interesting for the following reasons: the rarity of the case and difficult diagnosis; the risk of malignancy and catecholamine crisis; the presence of an inguinal hernia; and finally, the ureteral implication. Extra-adrenal pheochromocytoma is a rare differential diagnosis in patients presenting with a retroperitoneal tumor mass; however, with the described pitfalls, it is important to consider it.
